# Assessment of Venous Thromboembolism (VTE) Risk Evaluation and Compliance With Guidelines in Surgical Patients: A Clinical Audit at the Prince Osman Digna Referral Hospital

**DOI:** 10.7759/cureus.90550

**Published:** 2025-08-20

**Authors:** Mohamed Salah Ibrahim Ahmed, Abdulrahman Abbas Yusuf Mohammed, Awad Abdalla Widaa Mohamed, Ahmed Khalid Mohamed Ahmed, Ahmed N Mohamed, Mohammed Osman Ahmed Osman, Soudad A Shila, Mohey Aldien Ahmed Elamin Elnour, Mohamed Hamza Mohamed Bakheet, Abdulrahman Mohammed, Ashraf H Hassan, Elaf Osama Elsamani Altayb, Marwa Elaziz, Amal A Fadl, Matab A Abdalla, Suliman Saadeldeen, Mustafa Mohamed, Alnazeer Y Abdelbagi, Moazar Haytham Mawiya Merghani

**Affiliations:** 1 General Surgery, Prince Osman Digna Referral Hospital, Port Sudan, SDN; 2 Internal Medicine, University of Gezira, Wad Madani, SDN; 3 Internal Medicine, Dongola Specialized Hospital, Dongola, SDN; 4 General Surgery, Port Sudan Teaching Hospital, Port Sudan, SDN; 5 Critical Care Medicine, SEHA Salma Rehabilitation Hospital, Abu Dhabi, ARE; 6 Internal Medicine, The National Ribat University, Khartoum, SDN; 7 Medical Education, Hamad Medical Corporation, Doha, QAT; 8 Neurosurgery, University Hospitals Coventry and Warwickshire NHS Trust, Coventry, GBR; 9 General Surgery, University of Kassala, Kassala, SDN; 10 General and Colorectal Surgery, The Royal Oldham Hospital, Manchester, GBR; 11 Emergency, Dr. Sulaiman Al Habib Hospital, Riyadh, SAU; 12 Medicine and Surgery, Hamad Medical Corporation, Doha, QAT; 13 Cardiology, Rashid Hospital, Dubai, ARE; 14 General Surgery, Hamad Medical Corporation, Doha, QAT; 15 General Surgery, Managil Teaching Hospital, Managil, SDN; 16 Internal Medicine, Prince Osman Digna Teaching Hospital, Port Sudan, SDN; 17 Anatomy, University of Khartoum, Khartoum, SDN

**Keywords:** caprini score, prince osman digna referral hospital, quality improvement, surgical inpatients, thromboprophylaxis, venous thromboembolism

## Abstract

Background

Venous thromboembolism (VTE), encompassing deep vein thrombosis (DVT) and pulmonary embolism (PE), is a leading cause of avoidable hospital-acquired morbidity and mortality. Despite clear international guidelines from bodies such as the American College of Chest Physicians (ACCP) and the National Institute for Health and Care Excellence (NICE), compliance in surgical settings remains suboptimal. This clinical audit aimed to evaluate and improve the documentation and implementation of VTE risk assessment and prophylaxis among surgical patients.

Methods

A closed-loop clinical audit was conducted at Prince Osman Digna Referral Hospital, Port Sudan, Sudan. The study included retrospective and prospective data collection across two audit cycles, the first involving 50, and the second cycle, 47 randomly selected surgical inpatient records. Data were gathered using a standardized tool aligned with international guidelines. Interventions between the cycles included educational workshops, implementation of standardized assessment forms, visual aids, and feedback sessions. Data were analyzed using descriptive statistics and the Chi-square test to determine changes in compliance.

Results

In the first cycle (N = 50), compliance was poor: zero (0%) had Caprini scores documented, and 49 (98%) received no prophylaxis. Following targeted interventions, the second cycle (N = 47) showed significant improvement. Caprini score documentation increased to 39 (83%), early mobilization to 30 (63.8%), and LMWH administration to nine (19.1%). Use of TED stockings rose to eight (17%), while those receiving no prophylaxis dropped to seven (14.9%). Documentation of prophylaxis duration improved to 37 (78.7%), compared to one (2%) in the first cycle. Guideline-consistent management plans increased from one (2%) to 30 (65.2%), and education on VTE risk and prophylaxis rose from one (2%) to 20 (42.6%). All improvements were statistically significant (p < 0.05).

Conclusion

Targeted interventions led to substantial improvements in VTE risk assessment and prophylaxis practices. Standardized tools, clinician education, and systematic feedback proved effective in promoting adherence to evidence-based guidelines. Sustained efforts are essential to maintain compliance and enhance patient safety.

## Introduction

Venous thromboembolism (VTE), comprising deep vein thrombosis (DVT) and pulmonary embolism (PE), remains one of the leading and largely preventable causes of hospital-acquired morbidity and mortality worldwide [1]. Surgical patients, in particular, are at significantly higher risk due to multiple predisposing factors. The physiological stress of surgery, prolonged immobilization, vascular injury, and systemic inflammatory responses contribute to the activation of Virchow’s triad: venous stasis, endothelial damage, and hypercoagulability, which collectively increase the risk of thromboembolic events in this population [2].

The public health and economic burden of VTE is considerable. Globally, thousands of deaths each year are attributable to PE, and a large proportion of these cases are preventable with appropriate prophylactic strategies [3]. Beyond the acute phase, survivors often suffer from long-term complications such as post-thrombotic syndrome and chronic thromboembolic pulmonary hypertension. These sequelae not only compromise patients’ quality of life but also lead to increased healthcare utilization, prolonged hospital stays, and readmissions [4].

Robust evidence from randomized controlled trials and meta-analyses demonstrates that timely and appropriate pharmacological prophylaxis can reduce the incidence of hospital-acquired VTE by 50%-70% [5]. In recognition of this, several international bodies, including the American College of Chest Physicians (ACCP), the National Institute for Health and Care Excellence (NICE), and the American Society of Hematology (ASH), have developed evidence-based guidelines to optimize VTE prevention across different patient populations [6-8]. These guidelines recommend routine risk stratification, the use of validated scoring systems such as the Caprini model, and the implementation of tailored pharmacological and/or mechanical prophylaxis depending on the patient’s risk profile.

Despite the availability of these well-structured guidelines, real-world adherence remains suboptimal. Studies have shown that compliance is often undermined by gaps in knowledge, lack of awareness of available tools, inconsistent documentation, and absence of institutional protocols [1]. Furthermore, mechanical methods of prophylaxis, such as graduated compression stockings or intermittent pneumatic compression devices, are frequently underutilized or incorrectly applied.

Quality improvement initiatives have demonstrated that multifaceted approaches, especially those integrating education, audit-feedback loops, and decision support tools, are more successful in improving compliance and patient outcomes. For example, a study published in BMJ Quality & Safety highlighted that programs combining active education and clinical reminders can drive adherence rates close to 100%, whereas passive dissemination strategies often result in only modest improvements [1].

In low-resource settings and in facilities with high surgical volumes, the implementation of standardized VTE prevention practices is particularly challenging [9]. Therefore, structured audits can play a critical role in identifying existing practice gaps, promoting clinician accountability, and guiding interventions tailored to the local context.

This clinical audit was conducted to assess the comprehensiveness of VTE risk assessment and the appropriateness of prophylaxis measures among surgical inpatients at a referral hospital. Historically, VTE prevention practices at this hospital were suboptimal, with no structured risk assessment tools or standardized prophylaxis protocols in routine use, and reliable data on the incidence of VTE events were limited. The objectives of this audit were to evaluate current practices, enhance adherence to international guidelines, and ultimately reduce the incidence of preventable VTE-related complications and deaths in this vulnerable patient group.

## Materials and methods

Study design and setting

This clinical audit included both retrospective and prospective observational elements, organized as a closed-loop audit. The research was performed at Prince Osman Digna Referral Hospital, Port Sudan, Sudan - a tertiary care facility offering specialist surgical treatments. Data were obtained from patient records and hospital logs using a standardized audit instrument.

The evaluation was conducted using a pre-established questionnaire consistent with worldwide VTE prevention recommendations (see Appendix), including those from the NICE and the ACCP [6,7]. The main aim was to examine compliance with established protocols for DVT risk evaluation and prevention in surgical patients.

The first audit cycle occurred from October 2024 to January 2025, followed by an intervention phase that lasted for two months, and thereafter, a second prospective cycle from March 2025 to July 2025. Each cycle included a thorough assessment and documentation of patient records to assess adherence to audit requirements. Data collection and analysis were conducted using Google Forms (Google, Inc., Mountain View, CA, USA) for organized data input and descriptive statistical assessment.

Audit area and population

The clinical audit was conducted in the Surgical Department of Prince Osman Digna Referral Hospital, a tertiary referral center that provides specialized surgical services to a large and diverse patient population. The audit aimed to evaluate the documentation and implementation of VTE risk assessment and prophylaxis practices among surgical inpatients.

All adult patients admitted under the Surgical Department during the designated audit periods were considered for inclusion. The inclusion criteria required that patients must have undergone a surgical procedure during admission, and have complete documentation related to VTE risk assessment and prophylactic measures. Patient records were excluded if they were illegible, contained missing information regarding VTE assessment or prophylaxis, or if the admission did not involve any surgical intervention.

The audit was conducted in two cycles. The first cycle was performed retrospectively between October 2024 and January 2025, during which patient records, operative notes, prescription charts, and hospital logs were systematically reviewed to assess baseline VTE risk assessment and prophylaxis practices. Following this, a set of targeted interventions was implemented, including interactive educational sessions for surgical and nursing staff, visual reminders placed in wards and operating theaters, and the introduction of standardized VTE risk assessment and prophylaxis documentation tools. The second cycle was conducted prospectively from March 2025 to July 2025, to evaluate the impact of these interventions, with real-time monitoring and data collection to ensure accuracy and completeness. All data were assessed against international guidelines, including those from the ACCP and NICE, to determine adherence and identify ongoing gaps in practice [6,7].

Sample size and sampling technique

For analysis, 50 records from the first cycle and 47 from the second cycle were examined, a total of 97 patient records. In line with previous audits carried out in similar hospital settings, the sample size was decided upon taking into account the available resources and the viability of the project. This sample size was deemed sufficient to detect trends in compliance and quantify the effect of treatments, even though no formal power calculation was conducted. In both cycles, all available patient records meeting the inclusion criteria were included, with no exclusions applied. 

Data collection

The data collection used a systematic questionnaire formulated in accordance with worldwide VTE prevention standards, including those from NICE and ACCP [6,7]. The initial guideline components were examined and modified to align with the local clinical setting at Prince Osman Digna Referral Hospital, to guarantee practicality and pertinence. The modification procedure included elucidating definitions for certain audit criteria and excluding elements irrelevant to the available resources. The final instrument evaluated essential criteria, including the existence of recorded VTE risk assessments, the suitability of pharmacological or mechanical prophylaxis, and the recording of individual patient risk variables.

The audit included 97 patient records, with 50 examined in the first round and 47 in the second round. All surgical inpatients who satisfied the inclusion criteria were eligible for selection. Records were omitted if they were devoid of critical clinical information or related to non-surgical hospitalizations. A straightforward random sampling method was used to choose records for examination, guaranteeing a representative sample.

Data were gathered by a team of competent medical experts who participated in a standardization session to guarantee consistent interpretation of the checklist items. A preliminary evaluation of 10 records was performed before official data collection to enhance the procedure and augment inter-rater reliability. Data input was conducted using Google Forms, which automatically transferred replies into an electronic spreadsheet for further analysis. Each parameter was classified as “compliant” or “non-compliant,” with confusing entries adjudicated by agreement among the audit team. To ensure confidentiality, patient identities were eliminated before data input, and all data were kept on a secure, password-protected device, accessible only to the audit team.

Statistical analysis and data

The data collection used a systematic questionnaire formulated according to worldwide VTE prophylaxis recommendations, including those established by NICE and ACCP [6,7]. The initial guideline components were evaluated and modified to align with the local clinical setting at Prince Osman Digna Referral Hospital, to guarantee practicality and pertinence. This adaptation procedure included elucidating definitions for certain audit criteria and excluding elements irrelevant to the available resources. The final instrument evaluated essential aspects, including the existence of recorded VTE risk assessment, the suitability of pharmacological or mechanical prophylaxis, and the recording of patient-specific risk variables.

The audit included 97 patient records, with 50 examined for the first cycle and 47 for the second cycle. All surgical inpatients who fulfilled the inclusion criteria were eligible for selection. Records were omitted if they were devoid of critical clinical information or related to non-surgical hospitalizations. A simple random selection method was used to choose records for examination, guaranteeing a representative sample.

Data analysis was performed with Google Sheets and Microsoft Excel 2016 (Microsoft Corporation, Redmond, WA, USA). Descriptive statistics were used to encapsulate the data, with categorical variables represented as frequencies and percentages. The main outcome measure was the compliance rate for VTE risk assessment and prophylaxis during the two audit cycles.

The Chi-square (χ²) test was used to evaluate categorical data about compliance before and after the intervention. This test was suitable due to the categorical characteristics of the variables and the independence of the observations. The assumptions of the Chi-square test, including the minimum predicted cell counts, were confirmed before analysis. The threshold for statistical significance was established at a p-value of less than 0.05.

Missing data were addressed by designating a “non-compliant” status to any unrecorded or ambiguous parameter, thereby assuring analytical completeness and mitigating bias from case exclusion. Results were expressed as percentages for each parameter, and overall compliance was determined by dividing the total number of recorded parameters by the anticipated total, then multiplying by 100.

Audit cycles

First Cycle: Pre-intervention

The first audit cycle was performed retrospectively over three months, with the objective of establishing a baseline for adherence to VTE risk assessment and prophylactic protocols. Fifty patient records from the Surgical Department were examined using the modified audit instrument. The evaluated parameters included the documentation of VTE risk assessment, the prescription of pharmacological prophylaxis (e.g., low molecular weight heparin, or LMWH), and the use of mechanical prophylaxis, where warranted. Preliminary results revealed significant shortcomings in risk assessment and prophylactic execution, characterized by uneven documentation and frequent neglect of mechanical interventions despite evident clinical signs.

Intervention Phase

Subsequent to the analysis of the first cycle, a comprehensive intervention approach was executed to enhance compliance with recommendations. This included the implementation of a standardized VTE risk assessment and prophylaxis form to enhance documentation efficiency and maintain consistency in clinical practice. Educational workshops were held for surgical teams to underscore the significance of VTE prevention and adherence to guidelines, concentrating on the appropriate use of pharmacological and mechanical interventions. Visual aids, encapsulating essential procedures for risk assessment and prevention, were shown in wards and operating theaters to promote these practices. Moreover, regular feedback meetings were organized to tackle issues, clarify roles, and uphold responsibility among clinical personnel.

Second Cycle: Post-intervention

The second audit cycle was undertaken prospectively from March 2024 to July 2025, including a review of an additional 47 patient records from the same Surgical Department. This cycle sought to assess the efficacy of the treatments by juxtaposing compliance rates with those from the first cycle. The evaluated parameters were uniform with the first cycle to facilitate precise comparison. The results from this cycle indicated substantial improvements in documentation and prophylactic compliance subsequent to the execution of organized interventions.

Ethical considerations

Ethical approval for this clinical audit was obtained from the Institutional Review Board (IRB) of Prince Osman Digna Referral Hospital under reference number (2024/0076/A). As this was an audit of existing clinical practices and involved no direct patient contact, informed consent was not required. All data were anonymized prior to analysis to ensure patient confidentiality, and no identifiable information was included in the dataset. The audit was conducted in compliance with institutional policies and the principles outlined in the Declaration of Helsinki.

## Results

A total of 50 records were reviewed during the first audit cycle, and 47 records were assessed in the second cycle following the implementation of targeted interventions. Compliance with key parameters of VTE risk assessment and prophylaxis demonstrated significant improvement between the two cycles.

Patient demographics

The demographic characteristics of the patients in both audit cycles are presented in Table 1. The two cohorts were comparable in terms of age and sex distribution. In the first cycle, the mean age was 59.4 ± 14.2 years, with 54% being male. In the second cycle, the mean age was 61.1 ± 13.6 years, and 55.3% were male.

**Table 1:** Demographic Characteristics of Patients in the First and Second Audit Cycles The table summarizes the age and sex distribution of the patients included in both audit cycles. The demographic profiles were broadly similar between the two groups. SD, Standard Deviation

Characteristic	First Cycle (n = 50)	Second Cycle (n = 47)
Age, mean ± SD (years)	59.4 ± 14.2	61.1 ± 13.6
Male	27 (54%)	26 (55.3%)
Female	23 (46%)	21 (44.7%)

VTE risk assessment and prophylaxis compliance

One of the most notable improvements was in the documentation of VTE risk stratification using the Caprini scoring system. In the first cycle, none of the records (zero, 0%) included a documented Caprini score. In contrast, the second cycle showed 39 (83%) had this assessment documented, reflecting a substantial increase (p < 0.00001). This improvement is indicative of better risk identification practices and adherence to evidence-based guidelines.

Documentation of the prophylaxis plan also showed considerable enhancement. In the initial cycle, early mobilization was not documented in any of the reviewed cases (zero, 0%). However, in the second cycle, 30 (63.8%) included early mobilization as part of the management plan, representing a significant step toward implementing basic mechanical preventive measures. Pharmacological prophylaxis with LMWH also improved, increasing from only one (2%) in the first cycle to nine (19.1%) in the second cycle. Similarly, the use of thromboembolic deterrent (TED) stockings, which was absent in the first cycle (zero, 0%), was documented in eight (17%) post-intervention.

Crucially, the number of cases in which no prophylactic intervention was given decreased dramatically. In the first cycle, 49 (98%) received no form of VTE prophylaxis, while in the second cycle, this figure dropped sharply to seven (14.9%), demonstrating an 83.1% reduction in omission of preventive measures. This shift reflects improved awareness and application of appropriate interventions by the clinical team.

Another area of significant improvement was the documentation of the duration of the prophylaxis plan. Initially, only one (2%) had clear documentation of prophylaxis duration. After the intervention, this was recorded in 37 (78.7%), indicating a 76.7% increase. This documentation is vital for ensuring appropriate timing and continuation of VTE preventive therapy throughout the inpatient stay and perioperative period.

Moreover, adherence to recognized clinical guidelines improved considerably. Only one (2%) in the first cycle demonstrated full compliance with guideline-based VTE prevention, whereas in the second cycle, 30 (65.2%) met this standard. This 63.2% increase highlights the effectiveness of standardizing clinical practice and aligning it with internationally accepted protocols.

Patient education regarding VTE risks and the importance of prophylaxis was another domain that saw significant enhancement. In the first cycle, only one (2%) included documentation of VTE-related patient education. This rose to 20 (42.6%) in the second cycle, reflecting a 40.6% improvement. Educating patients is a critical step in reinforcing compliance and ensuring shared decision-making.

Collectively, these findings underscore the substantial impact of targeted interventions, such as educational workshops, standardized assessment forms, and clinical reminders, on improving the quality of care. The improvements seen across all audited parameters suggest that the implemented strategies were highly effective in enhancing both documentation practices and clinical performance in VTE prevention. Full results are detailed in Table 2.

**Table 2:** Comparison of VTE Risk Assessment and Prophylaxis Practices Before and After Intervention Across Two Audit Cycles at Prince Digna Referral Hospital This table presents a comparative analysis of VTE risk assessment and prophylaxis practices among surgical inpatients at Prince Digna Referral Hospital. The first audit cycle included 50 records reviewed prior to intervention, while the second cycle assessed 47 records following targeted quality improvement measures. The data reflect frequencies and percentages for each key parameter, including use of the Caprini score, documentation of prophylaxis plans, application of preventive measures (early mobilization, LMWH, TED stockings), adherence to clinical guidelines, and provision of patient education. Chi-square statistics and p-values are provided to illustrate the statistical significance of changes observed between the two cycles. VTE, Venous Thromboembolism; LMWH, Low Molecular Weight Heparin; TED, Thromboembolic Deterrent

Item		First Cycle (N = 50)	Second Cycle (N = 47)	Improvement	Chi-square	p-value
Caprini Score Was Calculated		0 (0%)	39 (83%)	+83%	73.54	< 0.00001
VTE Prophylaxis Plan Components	Early mobilization	0 (0%)	30 (63.8%)	+63.80%	41.99	< 0.00001
							LMWH	1 (2%)	9 (19.1%)	+17.10%	6.34	≈ 0.012
	TED stockings	0 (0%)	8 (17%)	+17%	8.56	≈ 0.003
	None	49 (98%)	7 (14.9%)	-83.1%	71.57	< 0.00001
	Duration of Prophylaxis Plan Clearly Documented		1 (2%)	37 (78.7%)	+76.70%	64.77	< 0.00001
Management Plan Consistent With Recognized Guidelines		1 (2%)	30 (65.2%)	+63.20%	49.29	< 0.00001
Patient Provided With Education on VTE Risk and Prophylaxis		1 (2%)	20 (42.6%)	+40.60%	23.08	< 0.00001

## Discussion

This clinical audit demonstrated significant improvements in the documentation and implementation of VTE risk assessment and prophylaxis among surgical patients at Prince Osman Digna Referral Hospital following targeted interventions. Overall compliance with guideline-based prophylactic measures increased substantially between the audit cycles. These findings highlight that focused educational efforts, standardized documentation tools, and regular feedback can effectively enhance adherence to established VTE prevention protocols in surgical settings.

One of the most significant findings in this audit was the dramatic increase in documentation of the Caprini risk assessment score, which improved from 0% in the first cycle to 83% in the second cycle. The Caprini score is a validated and widely used tool for stratifying surgical patients’ risk of VTE, allowing clinicians to tailor prophylaxis appropriately [10]. Accurate risk stratification is critical, as it guides decisions on pharmacological and mechanical prophylaxis, optimizing patient safety while minimizing complications such as bleeding. Previous studies have demonstrated that implementing formal risk assessment tools like the Caprini score improves adherence to prophylaxis guidelines and reduces the incidence of postoperative VTE [11]. The marked improvement observed in this audit likely reflects the effectiveness of the standardized assessment forms and targeted educational interventions introduced between cycles.

Early mobilization is a critical component of VTE prophylaxis in surgical patients and has been shown to significantly reduce the risk of thromboembolic events by promoting venous return and preventing blood stasis in the lower limbs [11]. In this audit, documentation of early mobilization increased substantially following the intervention, reflecting improved awareness and implementation of this simple yet effective measure. Current guidelines from organizations such as the ACCP and the NICE emphasize the importance of encouraging patients to mobilize as soon as clinically feasible after surgery to mitigate VTE risk [6,7]. Early mobilization is especially important in surgical populations, where immobility is a major risk factor for thrombosis.

Pharmacological prophylaxis with LMWH remains the cornerstone of VTE prevention in moderate- to high-risk surgical patients and has been extensively validated for efficacy and safety [12]. The audit showed an increase in LMWH administration, indicating better adherence to guideline recommendations. Additionally, the use of mechanical prophylaxis, such as TED stockings, also increased but remained suboptimal. Combining pharmacological and mechanical methods is recommended to maximize protection, particularly in patients at very high risk or with contraindications to anticoagulants [7]. Importantly, the marked decrease in patients receiving no prophylaxis demonstrates progress in closing critical gaps in patient care, consistent with evidence showing that omission of prophylaxis significantly increases VTE incidence and associated morbidity and mortality [13].

Specifying the duration of VTE prophylaxis is a vital aspect of patient management, as both under- and over-treatment can have significant consequences. Appropriate duration depends on the patient’s risk profile, type of surgery, and mobility status. Clinical guidelines emphasize the importance of clearly documenting the intended length of prophylaxis to ensure continuity of care and reduce the risk of recurrent thromboembolism or bleeding complications [6]. In this audit, documentation of the duration of prophylaxis improved notably after intervention, reflecting enhanced clinician awareness and adherence to protocols. Studies have shown that extended prophylaxis beyond hospital discharge is beneficial in high-risk patients, such as those undergoing major orthopedic or cancer surgeries, further underscoring the need for precise duration planning [14].

Clear documentation of the duration of VTE prophylaxis is essential for effective patient care. In this audit, we observed a notable improvement in specifying prophylaxis duration, reflecting better adherence to clinical protocols. This enhancement supports safer, more consistent management by reducing risks associated with both inadequate and excessive treatment durations.

Educating patients about their personal risk for VTE and the rationale behind prophylactic measures is a vital, yet often overlooked, aspect of thrombosis prevention. By improving awareness, patients become more engaged in their care and more likely to comply with both pharmacological and mechanical prophylaxis. Our findings showed a significant increase in documented patient education after the intervention, indicating a positive shift toward involving patients as active participants in their health management. Leading thrombosis societies emphasize that effective patient communication not only empowers individuals but also plays a crucial role in early identification of VTE symptoms, potentially reducing complications and improving long-term outcomes [15].

Several previous audits have reported similar challenges and improvements in VTE prophylaxis adherence after implementing structured interventions. For example, a closed-loop audit conducted in Ireland showed an increase in documented risk assessments from 2.7% to 66%, along with high compliance with LMWH use after targeted education and standardized forms were introduced [16]. Similarly, a Scottish audit reported LMWH adherence improving from 86% to 90% following educational interventions and visual reminders [17]. These findings corroborate our results, demonstrating that multifaceted strategies effectively enhance VTE prophylaxis practices in surgical settings. However, despite significant progress, some gaps remain, particularly in the documentation of patient education and the use of mechanical prophylaxis, which warrants ongoing quality improvement efforts.

The improvement rates observed in our audit are comparable to findings from other resource-limited settings. For instance, a Sudanese prospective audit reported that VTE and bleeding risk assessments improved from 0% in the first cycle to 100% in the second cycle, while adherence to guideline-based prophylaxis increased from 0% to 58.6% after targeted interventions [9]. These results, similar to our own, underscore the effectiveness of structured education and the introduction of standardized tools in driving rapid improvements in clinical practice, even in hospitals facing significant resource constraints.

While this audit demonstrated significant improvements in VTE prophylaxis practices, certain limitations should be acknowledged. The reliance on documentation for data collection means that some aspects of clinical care may have been underrepresented if not properly recorded. Additionally, the sample size, though sufficient to identify trends, may limit the generalizability of findings across different settings. The audit period and timing of intervention phases were constrained by logistical factors, which could influence the magnitude of observed changes. Despite these challenges, the use of standardized data collection tools and clear audit criteria helped mitigate bias, and ongoing monitoring is planned to sustain and further enhance adherence to VTE prevention protocols.

## Conclusions

This audit demonstrates that targeted interventions can substantially improve adherence to VTE prophylaxis guidelines at Prince Osman Digna Referral Hospital. Building on these gains, future efforts should focus on institutionalizing standardized risk assessment tools, strengthening staff training programs, and integrating continuous audit cycles into routine practice. Such measures will be critical to sustaining improvements, reducing the burden of preventable thromboembolic events, and advancing patient safety and quality of care in the long term.

## Appendices

VTE audit data collection form/questionnaire

The data collection form used in this audit included the following sections:

Patient Demographics

Age

Sex

VTE Risk Assessment

Documentation of Caprini score (Yes/No)

VTE risk level (low, moderate, high)

Prophylaxis Measures

Early mobilization implemented (Yes/No)

Pharmacological prophylaxis with low molecular weight heparin (LMWH) administered (Yes/No)

Mechanical prophylaxis using thromboembolic deterrent (TED) stockings (Yes/No)

Duration of Prophylaxis

Documentation of prophylaxis duration (Yes/No)

Guideline Adherence

Full compliance with evidence-based VTE prevention guidelines (Yes/No)

Patient Education

Documentation that VTE risk and prophylaxis were explained to the patient (Yes/No)

Additional Comments

Any other relevant notes or observations
